# Spatial variations of tritium concentrations in groundwater collected in the southern coastal region of Fukushima, Japan, after the nuclear accident

**DOI:** 10.1038/s41598-017-12840-3

**Published:** 2017-10-03

**Authors:** Koki Kashiwaya, Yuta Muto, Taiki Kubo, Reo Ikawa, Shinji Nakaya, Katsuaki Koike, Atsunao Marui

**Affiliations:** 1 Department of Urban Management, Graduate School of Engineering, Kyoto University, Kyoto, Japan; 2 Groundwater Research Group, Geological Survey of Japan, National Institute of Advanced Industrial Science and Technology, Tsukuba, Japan; 3 Department of Water Environment and Civil Engineering, Faculty of Engineering, Shinshu University, Nagano, Japan

## Abstract

Spatial variations in tritium concentrations in groundwater were identified in the southern part of the coastal region in Fukushima Prefecture, Japan. Higher tritium concentrations were measured at wells near the Fukushima Daiichi Nuclear Power Station (F1NPS). Mean tritium concentrations in precipitation in the 5 weeks after the F1NPS accident were estimated to be 433 and 139 TU at a distance of 25 and 50 km, respectively, from the F1NPS. The elevations of tritium concentrations in groundwater were calculated using a simple mixing model of the precipitation and groundwater. By assuming that these precipitation was mixed into groundwater with a background tritium concentration in a hypothetical well, concentrations of 13 and 7 TU at distances of 25 and 50 km from the F1NPS, respectively, were obtained. The calculated concentrations are consistent with those measured at the studied wells. Therefore, the spatial variation in tritium concentrations in groundwater was probably caused by precipitation with high tritium concentrations as a result of the F1NPS accident. However, the highest estimated tritium concentrations in precipitation for the study site were much lower than the WHO limits for drinking water, and the concentrations decreased to almost background level at the wells by mixing with groundwater.

## Introduction

The huge earthquakes on and after 11 March 2011, the ensuing Tsunami, and the accident at the Fukushima Daiichi Nuclear Power Station (F1NPS) wreaked enormous damage in Japan. The National Institute of Advanced Industrial Science and Technology in Japan initiated a research project “Study on risk evaluation of groundwater pollution” following the disaster. The project aims were to: elucidate the influence of the earthquake disaster on groundwater quality; understand hydrological properties and vulnerability of regional groundwater systems; and obtain information for optimizing groundwater use in coastal regions of the Tohoku district^1,2^. To realize these aims, sampling and chemical analyses of groundwater, including possible contaminants and environmental tracers to determine the groundwater flow regime, were conducted. A review by Steinhauser^3^ revealed that various radioactive nuclides, including tritium, have been emitted to the environment as a result of the F1NPS accident. Tritium is a radioactive isotope of hydrogen with a half-life of 12.32 years^4^, and it has both natural and anthropogenic origins. It is a suitable tracer for estimating water circulation because it becomes part of the water molecules and is geochemically conservative, thus allowing more straightforward interpretation than with other tracers^5^. Therefore, tritium concentrations in groundwater were analysed in this project.

There are few research articles that report on the behaviour of tritium emitted to the terrestrial environment as a result of the F1NPS accident. Kakiuchi *et al*.^6^ measured tritium concentrations in the free water of plant shoots and tree leaves collected soon after (March, April, July, and August 2011) the F1NPS accident. Higher tritium concentrations were obtained nearer to the F1NPS, and tritium concentrations in atmospheric moisture on 17 March 2011 were estimated at 5.6 kBq/L from concentrations in free water^6^. Matsumoto *et al*.^7^ reported tritium concentrations of precipitation collected until the end of May 2011 at six locations, at distances of 170 km to 700 km from the F1NPS. The highest value was 160 TU, from a sample collected at Tsukuba. The concentrations returned to background levels within 5 weeks. A regression model between atmospheric tritium activity and the distance from the F1NPS was constructed and 1.5 × 10^4^ Bq/m^3^ (1.3 × 10^5^ TU) was estimated as the atmospheric tritium activity at the F1NPS soon after the emission. Ueda *et al*.^8^ described tritium concentrations in river water collected in the Fukushima prefecture from 2011 to 2014. Concentrations in river water, collected at short time intervals at two rivers under base flow conditions and flood events, were higher in 2011 compared to background levels. The concentrations decreased with time and returned to levels similar to background levels in 2013. During a flood event in July 2011, tritium concentrations tracked discharge rate variations. However, the peak tritium concentration appeared later than the peak discharge rate in a flood event in September 2011. The differences in mean annual tritium concentrations for flood events and base flow conditions were indistinguishable after 2012. Ueda *et al*.^8^ interpreted this to be a result of water with high tritium concentrations infiltrating to deeper levels and being diluted by soil water or groundwater. In addition, river water collected once a year (from 2012 to 2014) from 16 rivers and one dam under base flow conditions also showed elevated tritium concentrations compared to background levels. Their mean annual concentration decreased consistently from 2012 to 2014. Yabusaki *et al*.^9^ reported on tritium concentrations for seven groundwater and spring water samples collected from the northern coastal region in Fukushima prefecture, and pointed out the possibility that the tritium concentration was elevated by recharge from precipitation with high tritium concentrations after the F1NPS accident.

As reviewed above, tritium concentrations in some types of water samples collected around the F1NPS were reported, but the impact of tritium emitted by the accident on groundwater was not systematically evaluated. This paper addresses this knowledge gap and describes tritium concentrations in groundwater samples collected from the coastal plains in southern Fukushima approximately one and a half years after the earthquake. The main aim is to identify the cause of the spatial variations in tritium concentrations in groundwater. Another environmental tracer, sulphur hexafluoride (SF_6_), was also analysed. It was correlated with tritium concentrations and compared with lumped parameter models (LPMs) to detect anomalies of tritium concentrations as discrepancies of the measured values from LPMs.

## Field campaigns and geological setting

**Figure 1:**
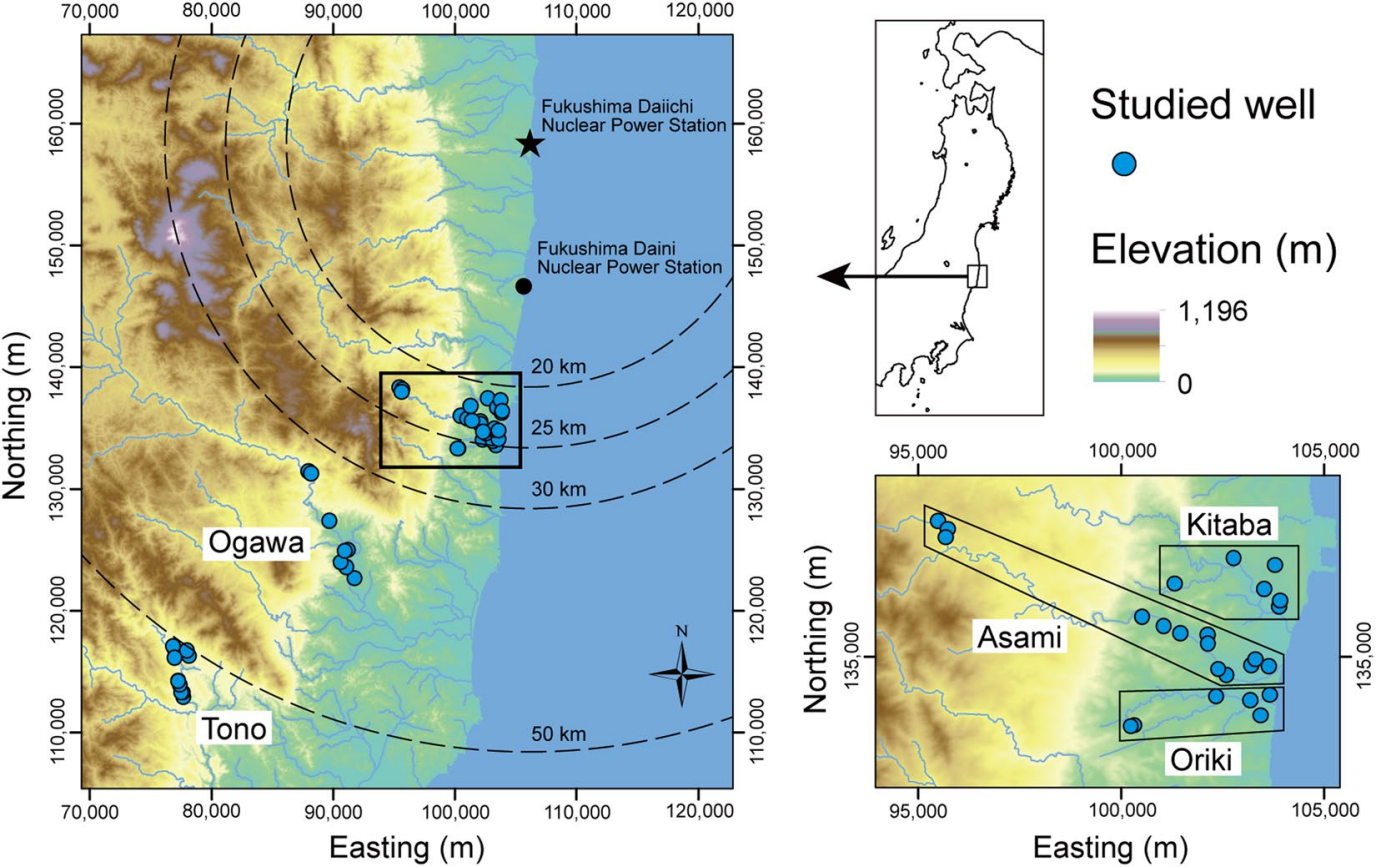
Location of the study area in northeastern Japan, water sampling sites, and grouping of wells into five areas. Circles represent the distance from the F1NPS (**a**). The coordinates in all the figures are JGD2000 and Japan Plane Rectangular CS IX. ASTER GDEM^32^ was used to draw the elevation map in this figure. ASTER GDEM is a product of NASA and METI. National Land numerical information (rivers data)^33^ offered by MLIT was used to draw the rivers in this figure.

**Figure 2:**
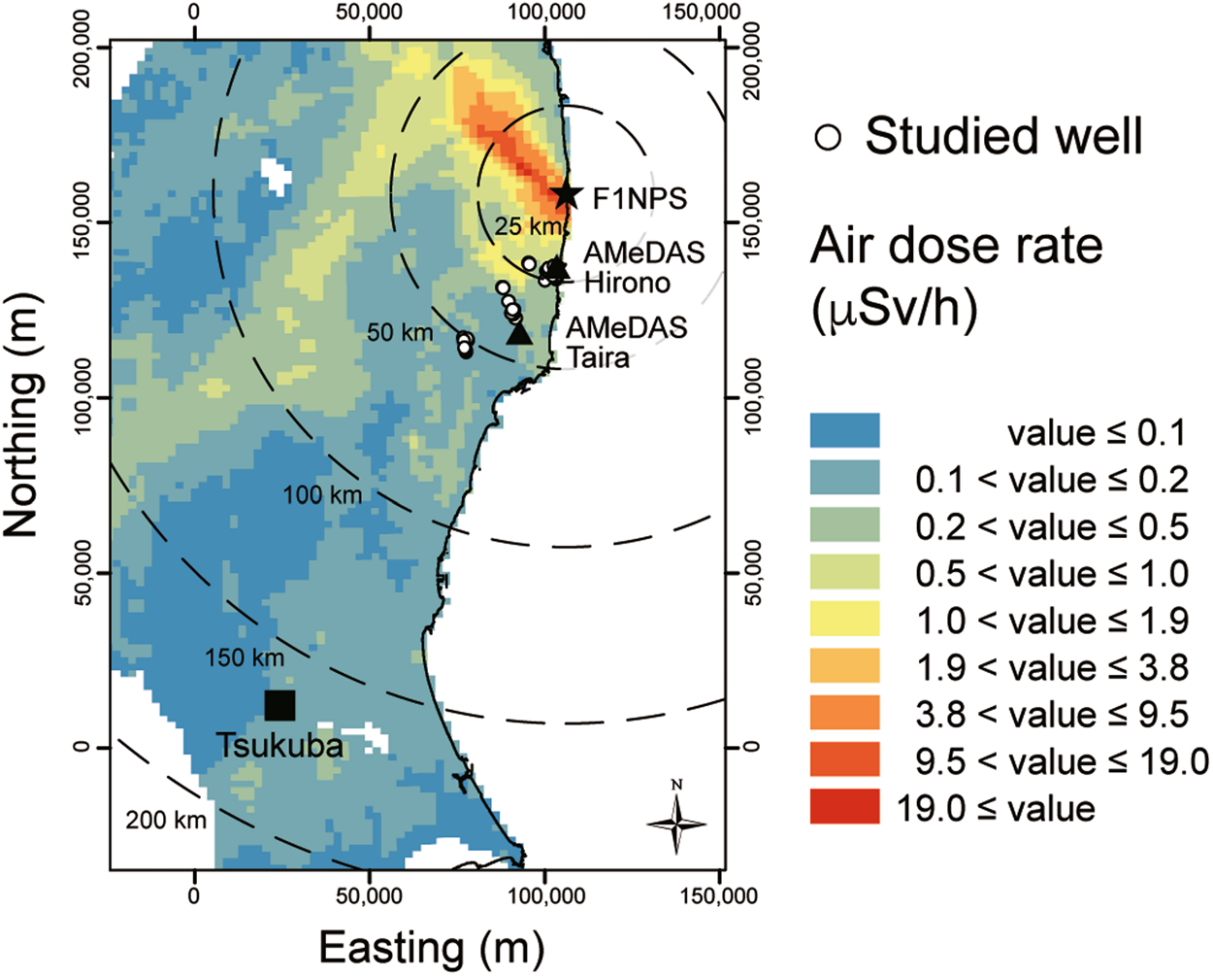
Air dose rates at 1 m above ground level. The air dose rates were estimated from the results of airborne monitoring conducted during 31 October to 28 December, 2012^10^. This map was drawn from the KMZ file available on the webpage of the Nuclear Regulation Authority, Japan^34^ using ArcGIS 10.3.1 (Esri, Inc.).

The study area comprises Iwaki City and Hirono Town in the southern part of the coastal region in the Fukushima Prefecture. Iwaki and Hirono are located to the south of the 20 km radius from the F1NPS (Fig. 1). Three field campaigns were conducted in 2012; groundwater samples were collected during 9 to 17 September, 21 to 27 October, and 11 to 17 November. Sampled wells were almost exclusively private wells used to draw water for domestic use, and were all pumped wells with a few exceptions. Fifty wells in total were sampled, and were grouped into five sub-areas by location: Tono (15 wells), Ogawa (8), Oriki (7), Asami (13), and Kitaba (7). A map of air dose rates at 1 m above the ground surface, drawn from results of airborne monitoring conducted from 31 October to 28 December 2012^10^, is shown in Fig. 2. A moderately high air dose rate zone extends from the F1NPS south-southwestward beyond Tsukuba. Matsumoto *et al*.^7^ reported the highest value of 160 TU for tritium concentration in precipitation collected at Tsukuba and estimated atmospheric tritium activity soon after the F1NPS accident. The wells investigated in the present study are distributed between the F1NPS and Tsukuba. It is helpful to consider the influence of tritium released through the accident on the groundwater environment around the F1NPS. The depth of the well bottom and groundwater level were measured using a flat tape water level meter. However, the water level meter could not be inserted at more than half of the wells because of the well structure, although the depth was obtained in most of these cases by interviewing the well owner.

The Tono wells were mostly located near the Iritono-gawa River, in alluvial sediments composed of sand gravel and sandy soil^11^. Except for three wells, their depths were equal to, or shallower than, 30 m. The Ogawa wells around the Natsui-gawa River are mainly located in three geological units: the Paleogene Shiramizu Group composed of conglomerate, sandstone, and shale, intercalated with coal seams^12^; Quaternary terrace deposits composed of gravel, sand, and mud; and alluvial sediments composed of gravel, sand, silt, clay, and surface soil^11^. Six of the eight wells were shallower than 30 m. The Oriki wells around the Oriki-gawa River were located in terrace deposits and alluvial sediments consisting of gravel, sand, and mud^13^. The depths of more than half of these wells were unknown. Known depths are shallower than 10 m. The Asami wells around the Asami-gawa River were mainly located in terrace deposits and alluvium sediments. Except for wells with an unknown depth, depths were equal to, or shallower than, 30 m. The Kitaba wells around Kitaba-gawa River were mostly located in terrace deposits composed of gravel, sand, and mud^13^. Their depths, where known, were less than 10 m.

In summary, most of the studied wells are shallow with an average depth of 14 m, and are distributed around the rivers. Permeable terrace deposits and alluvial sediments appear to be the main lithologies of the aquifers.

## Results

**Figure 3:**
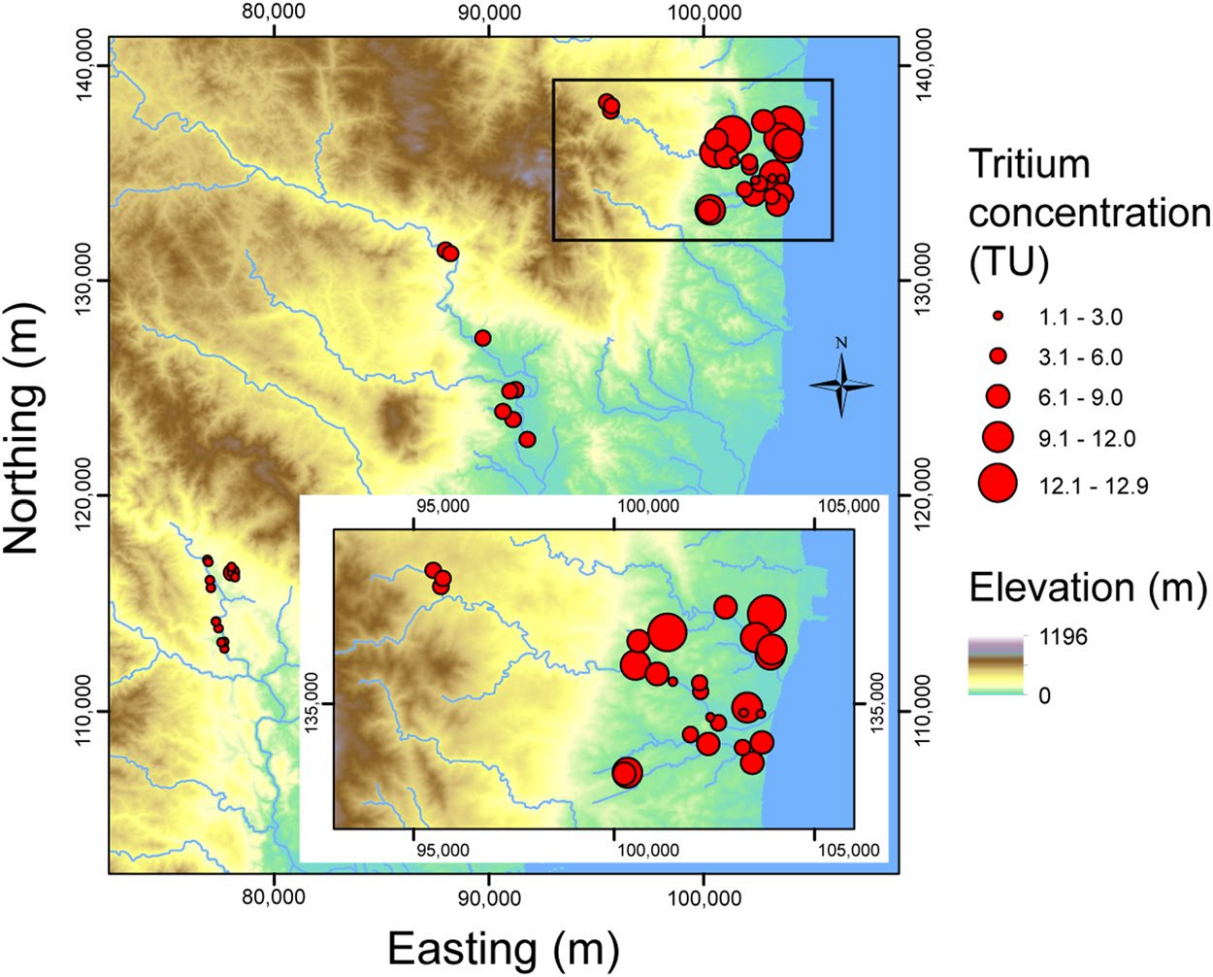
Groundwater tritium concentrations at the wells showing large spatial variations in the study area. ASTER GDEM^32^ was used to draw the elevation map in this figure. ASTER GDEM is a product of NASA and METI. National Land numerical information (rivers data)^33^ offered by MLIT was used to draw the rivers in this figure.

**Figure 4:**
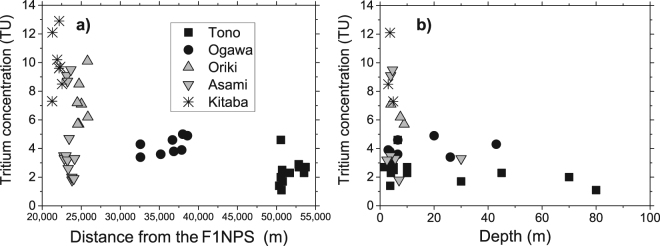
Correlation of groundwater tritium concentrations (Fig. 3) with distance from the F1NPS (**a**), and well depth (**b**). Data are classified according to the five areas in Fig. 1.

Tritium concentrations were measured by the liquid scintillation method, with an analytical error of ± 0.1 to 0.2 TU. A noteworthy feature of the results is that the concentration ranges differ according to the areas: 1.1 to 4.6 TU in the Tono area; 3.4 to 5.0 TU (Ogawa); 5.7 to 10.1 TU (Oriki); 1.8 to 9.5 TU (Asami); and 7.3 to 12.9 TU (Kitaba). Areal variations can also be discerned from the high concentrations located in the northeastern wells (Kitaba, Asami, and Oriki areas) and the low concentrations in the southwestern wells (Tono and Ogawa areas) (Fig. 3). The spatial trends are shown in more detail in scattergrams indicating the concentration variations with: the distance from F1NPS, and the depth of each well (Fig. 4). Wells of unknown depth were excluded in Fig. 4b. The highest concentrations were measured at the wells nearby F1NPS, and at depths shallower than 10 m. The Tono area is characterized by consistently low concentrations over a wide range of depths (1–80 m). The Ogawa area has slightly higher concentrations than the Tono area, but also over a wide range of depths. Compared with these two areas, the concentrations in Oriki, Asami, and Kitaba are higher and variable, but their wells are mostly shallow.

**Figure 5:**
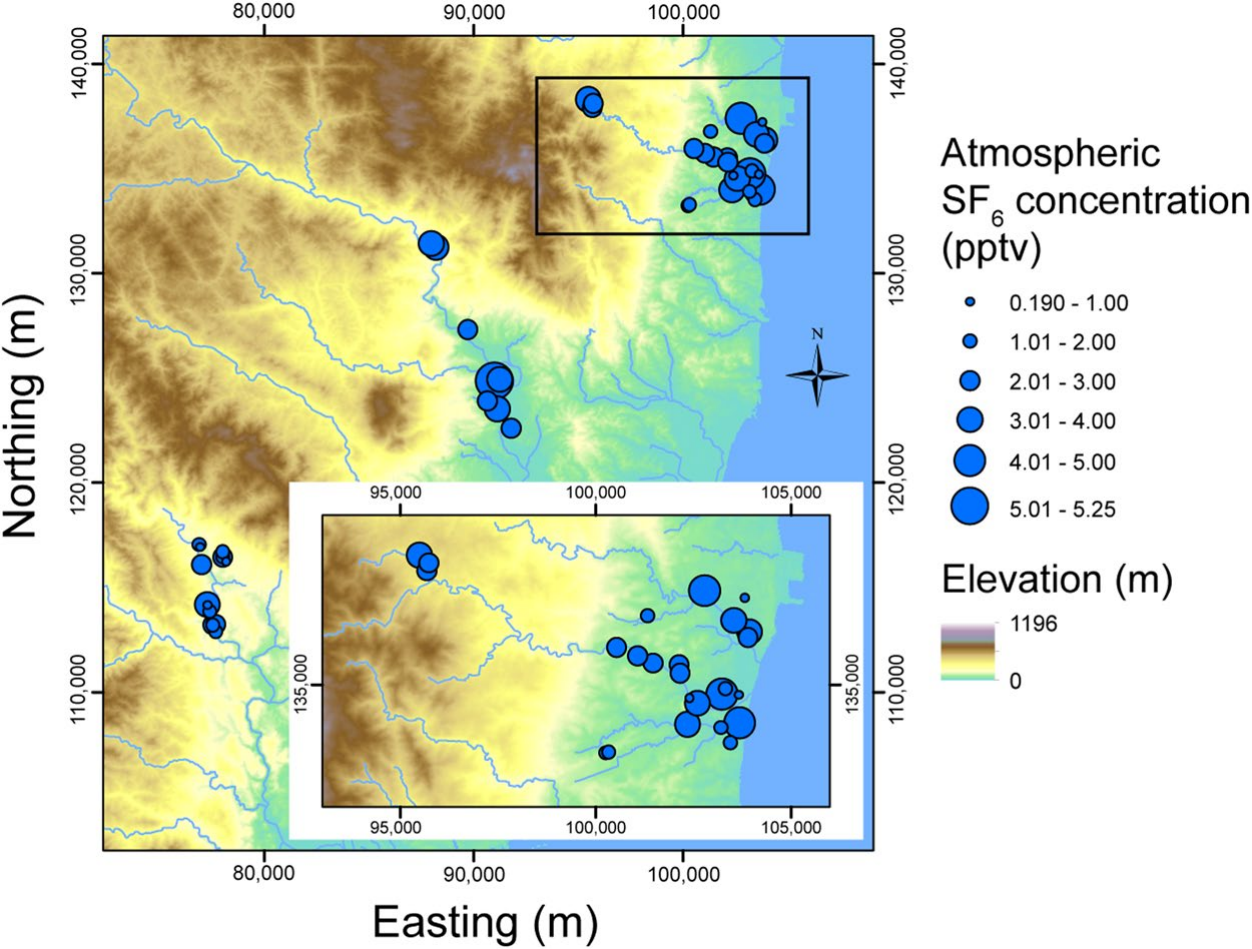
Atmospheric SF_6_ mixing ratios converted from the SF_6_ concentrations in groundwater at each well. ASTER GDEM^32^ was used to draw the elevation map in this figure. ASTER GDEM is a product of NASA and METI. National Land numerical information (rivers data)^33^ offered by MLIT was used to draw the rivers in this figure.

**Figure 6:**
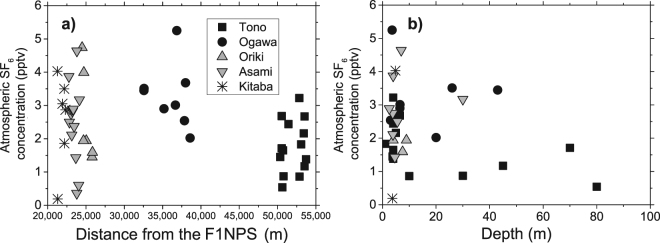
Correlation of atmospheric SF_6_ concentrations (Fig. 5) with distance from the F1NPS (**a**), and well depth (**b**). Data are classified according to the five areas in Fig. 1.

Figure 5 shows the regional variation in atmospheric mixing ratios of SF_6_, converted from SF_6_ concentrations in the groundwater samples. These maps indicate slightly lower mixing ratios in the Tono area (0.5 to 3.2 pptv and not detected at one well) and higher ratios in the Ogawa area (2.0 to 5.3 pptv). These ratios are similar to those in the Oriki, Asami, and Kitaba areas (Oriki: 1.5 to 4.7 pptv; Asami: 0.4 to 4.6 pptv; Kitaba: 0.2 to 4.0 pptv). The atmospheric mixing ratio converted from the groundwater concentration was higher than the present atmospheric mixing ratio at one well in Oriki (10.4 pptv) and one in Kitaba (55.9 pptv). Contamination of SF_6_ from sources other than atmospheric equilibrium was expected at these wells. Except for these two contaminated wells, the mixing ratios correspond to apparent residence times (Tono: 19 to 36 years and > 59 years; Ogawa: 9 to 24 years; Oriki: 12 to 28 years; Asami: 12 to 39 years; Kitaba: 15 to 44 years). Unlike the tritium concentrations, these mixing ratios do not correlate with the distance from the F1NPS, while a weak trend is observed only in the relationship with depth (Fig. 6). This correlation may originate from groundwater at depth generally having a longer residence time than shallow groundwater.

**Figure 7:**
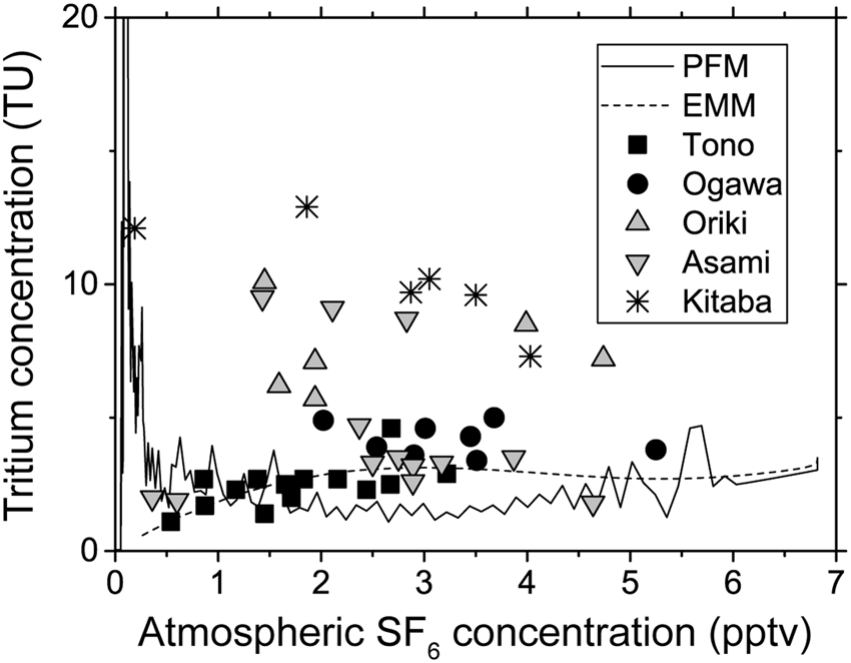
Relationship between groundwater tritium concentrations and atmospheric SF_6_ concentrations at wells grouped into five areas (Fig. 1), overlaid with the two lumped parameter models: PFM and EMM. The groundwater samples were collected during three field campaigns (9 to 17 September, 21 to 27 October, and 11 to 17 November).

Figure 7 shows the analytical results of tritium concentrations and SF_6_ mixing ratios together with the LPMs. The well data in the Tono, Ogawa, and part of the Asami areas can be closely fitted to the Exponential Mixing Model (EMM) curve. EMM correlates concentrations of environmental tracers by assuming the sample is a mixture of an infinite number of sub-parcels with an exponential age distribution^14^. This might apply to simple cases such as a spring from a homogeneous aquifer, receiving consistent recharge^14^. The consistency of the analytical results with the EMM model is reasonable considering the lithologies of the aquifers. In contrast, the well data from the northeastern areas, including Kitaba, Oriki, and Asami, show large deviations from the EMM model. Except for the two contaminated wells, mean residence times of the well groups were calculated using the EMM (Tono: 28 to 217 years and > 59 years; Ogawa: 16 to 48 years; Oriki: 18 to 70 years; Asami: 18 to 347 years; Kitaba: 21 to 725 years).

## Discussion

The spatial distribution of tritium concentrations was clearly discernible, and higher tritium concentrations were measured at wells nearer to the F1NPS and at wells with shallower depths (Fig. 4). Two mechanisms are possible causes of the high concentrations: 1) the influence of tritium emitted in the atmospheric nuclear bomb tests in the 20^th^ century, and 2) mixing of tritium rich precipitation resulting from tritium released to the atmosphere in the F1NPS accident.

The first mechanism is founded on the huge amount of tritium emitted by the atmospheric nuclear bomb tests conducted after 1950s, which elevated tritium concentrations in the hydrological cycle^5^. As a result of the radioactive decay of tritium, concentrations in recent precipitation have lowered to the background level before the bomb tests. In this study, higher tritium concentrations were measured in shallower wells (Fig. 4). This suggests the second mechanism, because these higher tritium concentrations at shallower depths are caused by the most recent precipitation, rather than the mixing of relatively old groundwater influenced by fallout from the bomb tests and distributed in the deeper parts of the aquifer.

The second mechanism assumes that the spatial distribution of tritium concentrations in groundwater was caused by tritium released from the F1NPS accident to the atmosphere. In the F1NPS reactors, tritium was mainly produced by the ternary fission of ^235^U and ^239^Pu; this was in addition to the neutron capture by deuterium and ^7^Li (n, an)^3^H reaction of lithium in the cooling water^15,16^. In the accident, tritium was released from the reactors damaged by the fuel meltdown and venting operation^17^. A tritium inventory on 11 March 2011 for the F1NPS reactor units 1, 2, and 3 were estimated to be in a total of 3.4 × 10^15^ Bq^18^. A portion of the tritium was considered to have been released to the atmosphere as tritiated water vapour and scavenged from the atmosphere as precipitation^8^. To strengthen the argument for the second mechanism, the influence of precipitation after the F1NPS accident on the groundwater tritium concentration is considered in conjunction with temporal and spatial variations in the tritium concentrations in precipitation after the accident.

Firstly, atmospheric tritium activity at the 25 km and 50 km points from the F1NPS soon after the accident were estimated based on an empirical relationship between the activity and the distance from the F1NPS^7^. These distances (25 km and 50 km) are equivalent to the distances of the northeastern wells and the southwestern wells from the F1NPS (Fig. 1). The activity values were determined to be approximately 16 Bq/m^3^ at the 25 km point, and 6 Bq/m^3^ at the 50 km point.

**Figure 8:**
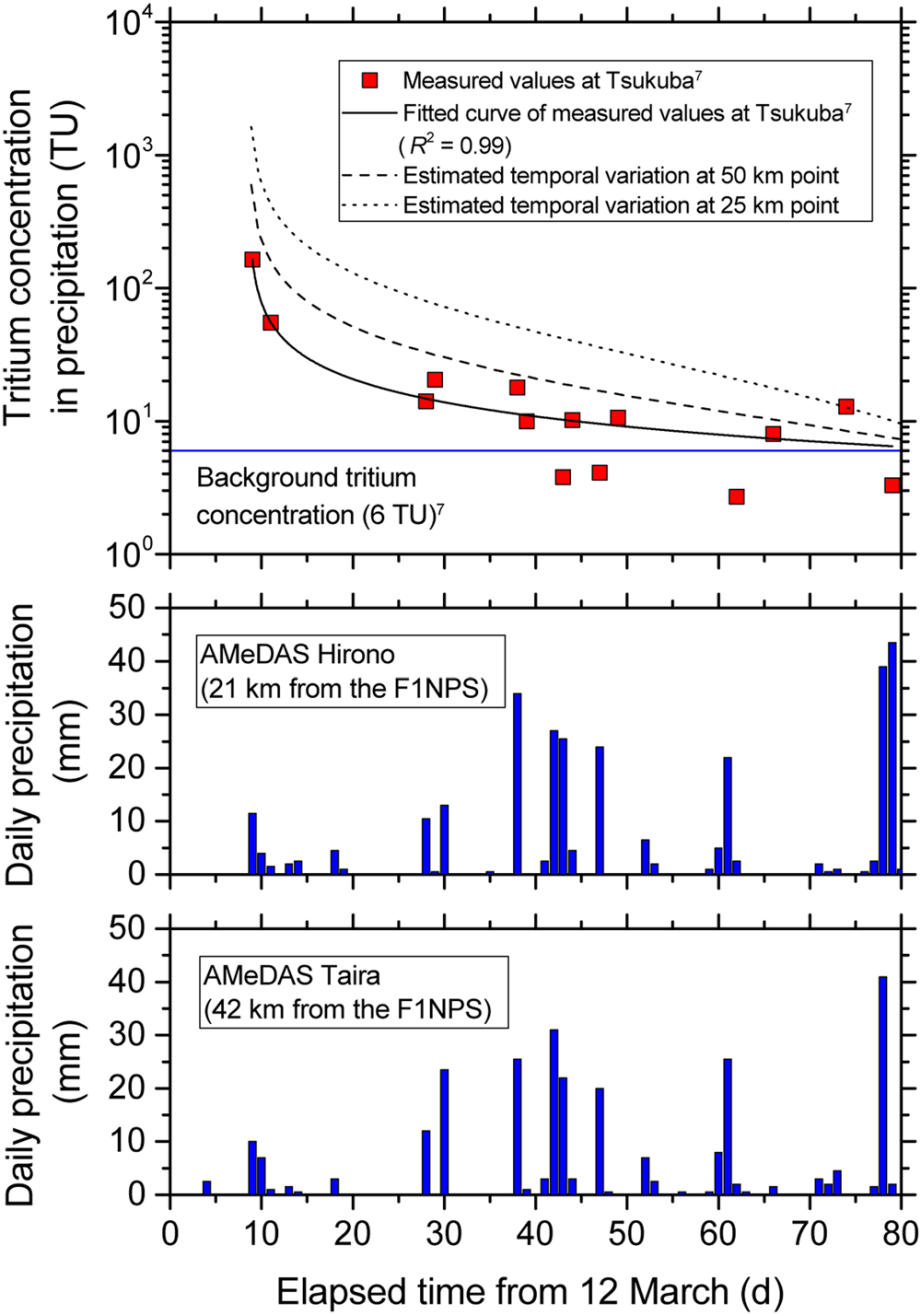
(**a**) Temporal changes in tritium concentrations in precipitation at a distance of 25 and 50 km from the F1NPS. The changes were estimated from the tritium concentrations measured at Tsukuba^7^ and its power law regression curve; (**b**) daily precipitation observed at the AMeDAS Hirono station^20^ (data missing between 14 and 20 March 2011); and (**c**) daily precipitation data at the AMeDAS Taira station^20^. (Precipitation of 2.5 mm on 16 March was not considered here because tritium concentrations at Tsukuba were measured after 21 March^7^ and the precipitation data at the Hirono station was not available for that date).

These atmospheric activities were converted to tritium concentrations in precipitation using a ratio of tritium concentration in precipitation to the atmosphere (washout ratio) of 1 × 10^4^, as estimated by Matsumoto *et al*.^7^ from tritium concentrations for the atmosphere and precipitation in Fukuoka, Japan^19^. The converted values (1342 TU at the 25 km point and 492 TU at the 50 km point) were used as precipitation concentrations for the time immediately after the accident, and time attenuation curves for precipitation tritium concentrations were estimated. Temporal changes in precipitation tritium concentrations at Tsukuba since 12 March 2011^7^ could be closely approximated by a power law (*R* ^2^ = 0.99) as shown in Fig. 8a. Tritium concentrations in precipitation at the 25 and 50 km points were assumed to follow this law. The curves of precipitation tritium concentration at the 25 and 50 km points, attenuating with time from the initial precipitation concentrations were reconstructed using the curve for Tsukuba (Fig. 8a). Background tritium concentration in precipitation, 6 TU^7^ was subtracted from the curve for Tsukuba (Fig. 8a) and the increment from the background level was linearly scaled as the obtained curves attenuated from the initial concentrations (1342 TU at the 25 km point and 492 TU at the 50 km point). The background tritium concentration was added back to the scaled increments and the temporal change curves of precipitation tritium concentration at the 25 and 50 km points were estimated.

As mentioned above, tritium concentrations in precipitation retuned to background levels in the 5-week period following the F1NPS accident^7^. Total precipitation amounts measured in this period at the adjacent observation stations of the Automated Meteorological Data Acquisition System (AMeDAS), maintained by the Japan Meteorological Agency, were 51.5 (Hirono) and 61.0 mm (Taira)^20^. The Hirono station and the Taira station are located 21 km and 42 km from the F1NPS (Fig. 2), respectively, and are thus comparable to the northeastern wells and southwestern wells. The tritium concentrations in precipitation in the 5-week period was calculated as a weighted mean from the tritium concentrations estimated using the attenuation curves and daily precipitation data at Hirono and Taira AMeDAS stations. The results obtained for the weighted mean precipitation tritium concentrations were 433 and 139 TU at the 25 and 50 km points, respectively.

The influences of the precipitation tritium concentrations on the tritium concentrations in groundwater at the wells were estimated with a simple mixing model. Tritium concentrations in the precipitation in the 5-week period after the accident and background levels for groundwater were used in the model. Tritium concentrations in precipitation before the F1NPS accident were estimated as 6 TU at Tsukuba^7^, and 0.1 to 1.5 Bq/L (0.8 to 13 TU) at Chiba from 2011 to 2014 (a mean of 0.4 ± 0.2 Bq/L (3 ± 2 TU)^8^) according to the Nuclear Regulation Authority^21^. These values are comparable to natural source tritium concentration in precipitation around the study area, obtained from simulations using Atmospheric General Circulation Models^22^. The median tritium concentration measured in the southwestern wells was 2.7 TU. This was similar to the mean tritium concentrations in precipitation at Chiba. The values for the southeastern wells were likely to be consistent with the EMM, and the highest tritium concentration is approximately 3 TU in the EMM (Fig. 7). Therefore, the background tritium concentration of groundwater in the study wells was considered to be 3 TU. The mixing model uses the tritium values for all the precipitation recharging wells during the 5-week period after the accident, and the ambient groundwater tritium values. The mixing ratio between precipitation and groundwater depends on groundwater levels in the wells. The mean value of water depth in the wells (2.2 m) was used in the calculation. The tritium concentrations in groundwater after mixing were then calculated as:

1 $${C}_{g}=\frac{{C}_{p}{a}_{p}+{C}_{b}(2200-{a}_{p})}{2200}$$

where *C*_g_ is the tritium concentration in groundwater after mixing; *C*_p_ is the tritium concentration of precipitation in the 5-week period after the F1NPS accident; (433 TU and 139 TU at the 25 km and 50 km points from the F1NPS); *a*_p_ is the precipitation amount in the 5-week period (51.5 mm at Hirono AMeDAS station and 61.0 mm at Taira AMeDAS station); and *C*_b_ is the background tritium concentration of groundwater (3 TU). The estimated tritium concentrations of groundwater were 13 TU and 7 TU at the 25 and 50 km points, respectively. These calculated concentrations are comparable to the measured concentrations in the study area. The spatial variation in groundwater tritium concentrations can be attributed to mixing by precipitation with high tritium concentrations originating from the F1NPS accident.

The discrepancy between the measured values and the LPM values could also be explained by the mixing of groundwater with precipitation with high tritium concentrations. After the Chernobyl accident, SF_6_ mixing ratios for groundwater, with apparent residence times that implied recharge around the time of the accident, largely exceeded the present atmospheric mixing ratio^23,24^. SF_6_ was considered to have been released by the explosion from electric components used in the power plant^23,24^. Contamination of such accidentally released SF_6_ also causes a discrepancy between the modelled and the measured values. In contrast, the SF_6_ mixing ratios of almost all the wells in the present study were clearly lower than the present atmospheric mixing ratio. In addition, the relationship between the SF_6_ mixing ratio and the distance from the F1NPS was not clear. Therefore, the influence of SF_6_ released through the F1NPS accident was presumably much less than that of the Chernobyl accident.

The estimated highest tritium concentrations in the precipitation was 1342 TU at the 25 km point on 21 March. This accidentally emitted tritium may be retained and detected as a peak of tritium concentration in the aquifers. However, the highest concentration in the precipitation (1342 TU) is much lower than the limit for drinking water (8.5 × 10^5^ TU) set by the World Health Organization^25^. Precipitation with high tritium concentrations that occurred soon after the accident was mixed and diluted with groundwater and, as a result, tritium concentrations have dropped to near background levels at the studied wells.

## Method

In the field campaigns, groundwater was pumped from wells and its pH, oxidation-reduction potential, electrical conductivity, and dissolved oxygen concentration were monitored. When these parameters became stable, samples for tritium and SF_6_ analyses were collected. The main dissolved components, trace elements, hydrogen and oxygen isotopes, radioactive caesium, and radon were also analysed but their results are not discussed here.

Tritium concentrations were measured using the liquid scintillation counter method. First, the sample was distilled and tritium in the sample was enriched by electrolysis. Then, with the addition of an emulsion scintillator, tritium in the electrolysis solution was detected using a low background scintillation counter. SF_6_ concentrations were measured with a purge and trap gas chromatograph-electron capture detector (GC-ECD) system. SF_6_ in the sample was extracted by bubbling with high purity nitrogen gas and recovered in a trap cooled with dry ice and ethanol. Trapped SF_6_ was liberated by heating the trap, and after trapping it was once again introduced to the GC-ECD for detection. SF_6_ quantities were calculated from the area of the SF_6_ peak in a chromatogram and from a calibration curve, which was converted to SF_6_ concentrations in the groundwater sample and then to the mixing ratio of SF_6_ in the atmosphere that was at equilibrium with the water at recharge.

Analytical results of tritium concentrations and atmospheric mixing ratios of SF_6_ were compared with LPMs^26–30^ that can estimate correlations of concentrations between multiple tracers in groundwater. These models are based on highly simplified hydrogeological models. The study area lacked details on hydrogeological structures. Therefore, the Piston Flow Model (PFM) and Exponential Mixing Model (EMM) were employed to characterize regional differences in the concentrations of the environmental tracers. These models are dependent only on mean transit time^29^ and are not controlled by other model parameters. The PFM assumes a single age for the sample water^14,29^ and corresponds to flow in confined aquifers^27,30^, while the EMM assumes the mixing of water with an exponential residence time distribution^14,27^ and can describe mixing of groundwater in pumped shallow groundwater and water discharge from springs in fissured aquifers^30^. Both models were run using Tracermodel1^14^, and input functions of temporal changes in precipitation tritium concentrations and SF_6_ mixing ratios in the atmosphere^31^. Several datasets were combined for the temporal change in tritium for the input function: the Global Network of Isotopes in Precipitation by the International Atomic Energy Agency (precipitation in Washington D.C. before 1961, Tokyo during 1961 to 1978, and Ryori during 1979 to the first half of 1986 and during 1998 to 2006); and the NIRS Environmental Tritium Survey Data Base of the National Institute of Radiological Sciences (for Chiba from the latter half of 1986 to 1997). Tritium concentrations after 2006 were assumed to be the same as in 2006. The SF_6_ input function used the atmospheric mixing ratio for North America compiled by the Reston Groundwater Dating Laboratory of the U.S. Geological Survey.
